# THE LONG ARMS OF CHILDHOOD INTELLIGENCE AND EDUCATION ON TERMINAL DECLINE: EVIDENCE FROM LOTHIAN BIRTH COHORT 1921

**DOI:** 10.1093/geroni/igz038.2311

**Published:** 2019-11-08

**Authors:** Dorina Cadar, Dorina Cadar, Annie Robitaille, Alison Pattie, Ian J Deary, Graciela Muniz Terrera

**Affiliations:** 1 University College London, London, United Kingdom; 2 Behavioural science and Health, University College London, London, England, United Kingdom; 3 Department of Psychology, Université du Québec à Montréal, montreal, Quebec, Canada; 4 School of Philosophy, Psychology and Language Sciences, university of Edinburgh, edinburgh, Scotland, United Kingdom; 5 Centre for Cognitive Ageing and Cognitive Epidemiology, University of Edinburgh, Edinburgh, Scotland, United Kingdom; 6 Centre for Clinical Brain Sciences, University of Edinburgh, Edinburgh, Scotland, United Kingdom

## Abstract

We investigated the heterogeneity of cognitive trajectories at the end of life by assigning individuals into groups according to their cognitive trajectories prior to death. Data were from the Lothian Birth Cohort of 1921. Growth mixture modelling was employed to identify groups of individuals with similar trajectories on the Mini-Mental State Examination in relation to time to death, accounting for childhood intelligence, education, hypertension, diabetes and cardiovascular disease. Two distinct groups of individuals (classes) were identified: a smaller class (18%) of individuals whose MMSE scores dropped linearly with about 0.5 points per year, and a larger group (82%) with stable scores across the study period. Childhood intelligence was associated with an increased probability of belonging to the stable class of cognitive functioning prior to death. These findings support a protective role of childhood intelligence, a marker of cognitive reserve, against the loss of cognitive function prior to death.

